# Discovering Subgroups of Children With High Mortality in Urban Guinea-Bissau: Exploratory and Validation Cohort Study

**DOI:** 10.2196/48060

**Published:** 2024-04-09

**Authors:** Andreas Rieckmann, Sebastian Nielsen, Piotr Dworzynski, Heresh Amini, Søren Wengel Mogensen, Isaquel Bartolomeu Silva, Angela Y Chang, Onyebuchi A Arah, Wojciech Samek, Naja Hulvej Rod, Claus Thorn Ekstrøm, Christine Stabell Benn, Peter Aaby, Ane Bærent Fisker

**Affiliations:** 1 Section of Epidemiology Department of Public Health University of Copenhagen Copenhagen Denmark; 2 Bandim Health Project, INDEPTH Network Bissau Guinea-Bissau; 3 Bandim Health Project, Research unit Odense Patient Data Explorative Network (OPEN), Department of Clinical Research Odense University Hospital and University of Southern Denmark Odense Denmark; 4 Novo Nordisk Foundation Center for Basic Metabolic Research University of Copenhagen Copenhagen Denmark; 5 Department of Environmental Medicine and Climate Science Icahn School of Medicine at Mount Sinai New York, NY United States; 6 Institute for Climate Change, Environmental Health, and Exposomics Icahn School of Medicine at Mount Sinai New York, NY United States; 7 Department of Automatic Control Lund University Lund Sweden; 8 Danish Institute for Advanced Study University of Southern Denmark Odense Denmark; 9 The Interdisciplinary Centre on Population Dynamics University of Southern Denmark Odense Denmark; 10 Department of Epidemiology Fielding School of Public Health University of California, Los Angeles Los Angeles, CA United States; 11 Department of Statistics and Data Science College of Letters and Science University of California, Los Angeles Los Angeles, CA United States; 12 Research Unit for Epidemiology Department of Public Health University of Aarhus Aarhus Denmark; 13 Department of Artificial Intelligence Fraunhofer Heinrich Hertz Institute Berlin Germany; 14 Department of Electrical Engineering and Computer Science Technical University of Berlin Berlin Germany; 15 Berlin Institute for the Foundations of Learning and Data Berlin Germany; 16 Section of Biostatistics Department of Public Health University of Copenhagen Copenhagen Denmark

**Keywords:** child mortality, causal discovery, Guinea-Bissau, inductive-deductive, machine learning, targeted preventive and risk-mitigating interventions

## Abstract

**Background:**

The decline in global child mortality is an important public health achievement, yet child mortality remains disproportionally high in many low-income countries like Guinea-Bissau. The persisting high mortality rates necessitate targeted research to identify vulnerable subgroups of children and formulate effective interventions.

**Objective:**

This study aimed to discover subgroups of children at an elevated risk of mortality in the urban setting of Bissau, Guinea-Bissau, West Africa. By identifying these groups, we intend to provide a foundation for developing targeted health interventions and inform public health policy.

**Methods:**

We used data from the health and demographic surveillance site, Bandim Health Project, covering 2003 to 2019. We identified baseline variables recorded before children reached the age of 6 weeks. The focus was on determining factors consistently linked with increased mortality up to the age of 3 years. Our multifaceted methodological approach incorporated spatial analysis for visualizing geographical variations in mortality risk, causally adjusted regression analysis to single out specific risk factors, and machine learning techniques for identifying clusters of multifactorial risk factors. To ensure robustness and validity, we divided the data set temporally, assessing the persistence of identified subgroups over different periods. The reassessment of mortality risk used the targeted maximum likelihood estimation (TMLE) method to achieve more robust causal modeling.

**Results:**

We analyzed data from 21,005 children. The mortality risk (6 weeks to 3 years of age) was 5.2% (95% CI 4.8%-5.6%) for children born between 2003 and 2011, and 2.9% (95% CI 2.5%-3.3%) for children born between 2012 and 2016. Our findings revealed 3 distinct high-risk subgroups with notably higher mortality rates, children residing in a specific urban area (adjusted mortality risk difference of 3.4%, 95% CI 0.3%-6.5%), children born to mothers with no prenatal consultations (adjusted mortality risk difference of 5.8%, 95% CI 2.6%-8.9%), and children from polygamous families born during the dry season (adjusted mortality risk difference of 1.7%, 95% CI 0.4%-2.9%). These subgroups, though small, showed a consistent pattern of higher mortality risk over time. Common social and economic factors were linked to a larger share of the total child deaths.

**Conclusions:**

The study’s results underscore the need for targeted interventions to address the specific risks faced by these identified high-risk subgroups. These interventions should be designed to work to complement broader public health strategies, creating a comprehensive approach to reducing child mortality. We suggest future research that focuses on developing, testing, and comparing targeted intervention strategies unraveling the proposed hypotheses found in this study. The ultimate aim is to optimize health outcomes for all children in high-mortality settings, leveraging a strategic mix of targeted and general health interventions to address the varied needs of different child subgroups.

## Introduction

Child mortality in Guinea-Bissau has decreased significantly over the past 40 years but is still unacceptably high (1 in 13 children dying before the age of 5 years in 2021 [1]). Thus, there is a constant need to design relevant interventions to reduce mortality [2,3]. In particular, identifying subgroups of children at high risk of dying may inform targeted preventive or risk-mitigating interventions to supplement population-wide approaches [4,5].

To identify actionable points for interventions to prevent or mitigate risk, we want to document the fuller causal structure. This spans distal causes such as social and economic conditions, legal rights, and welfare policies to immediate causes such as congenital malformations or infectious agents [6]. However, obtaining high-quality data on these factors can be challenging, particularly in low-income countries. One potential data source is Health and Demographic Surveillance Systems (HDSS), which collect individual-level data on demographics and health for a portion of the population [7].

In this exploratory study, we used HDSS data from urban Bissau, the capital of Guinea-Bissau, to identify subgroups of children at high risk of dying before 3 years of age. We analyzed data from 2003 to 2019, where the birth years from 2003 to 2011 were used to identify risk factors and high-risk groups, which we then tested in the birth years from 2012 to 2016. This allowed us to focus on factors consistently associated with high mortality over time. To do this, we used 3 different types of analyses, that are, spatial analysis to map child mortality in specific areas, regression analysis to identify single risk factors associated with high mortality, and a machine learning model to identify multifactorial risk groups. By integrating these approaches, we aimed to discover subgroups of children with high mortality without being limited to prior hypotheses [8]. Such discoveries are necessary for developing new hypotheses and identifying interventions to reduce child mortality.

## Methods

### Study Population and Follow-Up

The study population included children living in Bissau, the capital city of Guinea-Bissau. All the children were part of the HDSS Bandim Health Project [9] and were seen by data collectors within the first 6 weeks of life. Children under 3 years of age are routinely visited every 3-4 months to collect vital and health information. Many recorded child deaths in this population are due to infectious diseases such as respiratory infections, malaria, and diarrhea [10,11].

Follow-up for this study began at 6 weeks of age to ensure that a sufficient proportion of children had their baseline information recorded. Children, who died before 6 weeks of age or did not have complete baseline information, were excluded from the study (30,441/51,446, see flowchart in Figure S1 in Multimedia Appendix 1). To account for the potential selection bias caused by migrating children, inverse probability of censoring weights (IPCW) was used in all analyses and presented results (Multimedia Appendix 2) [12].

### Baseline Information

**Figure 1:**
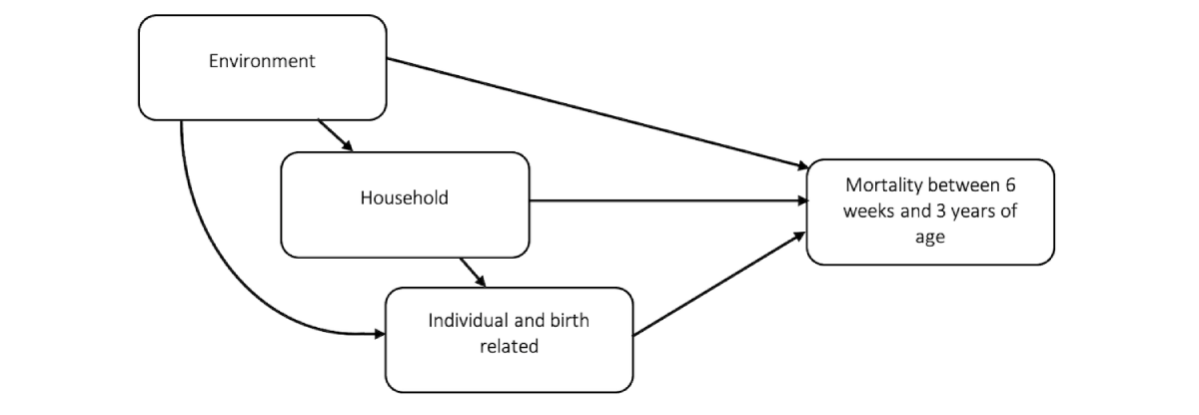
Flowchart of the assumed causal structure.

To identify relevant factors for child mortality, available baseline information was divided into environmental, household, and individual and birth domains. Figure 1 depicts the assumed causal structure [12] or the data-generating process linking these domains. Operational definitions of the variables and a visualization of their pairwise associations can be found in Table S1 and Figure S2 in Multimedia Appendix 1, respectively.

### Analyses

A temporal split of the data, rather than a random split, was used in this study. This allowed us to determine whether identified subgroups consistently had a higher mortality risk across different time periods and thus, may be relevant subgroups for future intervention. The sample size allowed us to divide the data into 2 cohorts. The temporal validation cohort also allowed us to test the robustness of our findings, as there have been various changes over time that may have affected child mortality, such as a significant decline in respiratory infections [11,13].

To describe the temporal trends in child mortality, the Kaplan-Meier estimator was used to calculate overall risks and risks split by age, accounting for censoring during follow-up. All subsequent analyses excluded censored children and used IPCW to adjust for selection bias.

We conducted the following 3 analyses to investigate the association between the baseline information collected before 6 weeks of age and mortality during the entire follow-up period (from 6 weeks to 3 years). All association measures were reported as mortality risk difference (MRD), with 0 indicating no difference between the compared subgroups. The MRD was expressed as a percentage (ie, the difference in deaths per 100 children). All estimates in the results section are adjusted mortality risk differences (aMRD).

### Spatial Analysis

We examined whether certain residential areas had a higher mortality risk than others by mapping the children’s households at baseline and moving a sliding window (250 m × 250 m) 10 m at a time to visualize the mortality risk across the study area. Estimates were only presented when at least 100 children were included within the sliding window to avoid small cell sizes. The estimates were adjusted for the linear effect of birth year, as child mortality has approximately decreased linearly by birth year (Figure S3 in Multimedia Appendix 1).

### Single Risk Factors

We used generalized linear regressions to investigate the associations between single factors and a higher risk of child mortality. Adjustments were made according to the assumed causal structure (depicted in Figure 1) by blocking the common causes in higher-order domains using the backdoor criterion [12].

### Multifactorial Risk Groups

We applied the Causes of Outcome Learning approach [14,15] to identify vulnerable subgroups with a combination of baseline information that was associated with a higher risk of child mortality. This causal inference-inspired machine learning approach has been optimized to prevent causal biases such as confounding by calendar time and collider bias, which could occur in other supervised clustering approaches. Details of the implementation of the Causes of Outcome Learning approach can be found in Multimedia Appendix 3. Since this approach is optimized for interactions in subpopulations, it is expected to find other patterns than the first-order linear regression which averages across the entire population.

### Summarization and Causal Modeling

To summarize the findings from the 3 analyses, key statistics such as prevalence, crude risks, and identification of synergistic associations [16] (where the risk from simultaneous exposure to multiple factors is greater than the sum of the individual risks) were calculated. Adjusted risk differences were determined using causal modeling (targeted maximum likelihood estimation [TMLE] [17]) for the defined subgroups compared to all other children. The probability of the estimates from the hypothesis-generating and temporal validation cohorts being similar was also calculated. In addition, a combined estimate for both cohorts was obtained to estimate the population attributable fraction (PAF) [18], which represents the fraction of all mortality that would be prevented if the causal exposure of interest was removed. The analyses were conducted using R (version 4.2; R Core Team), and some sentences were revised using ChatGPT (OpenAI) to improve clarity.

### Ethical Considerations

The study does not include biologically, physically invasive, or potentially dangerous procedures. The HDSS collection of data is at the request of the Ministry of Health, Guinea-Bissau.

## Results

### Overview

A total of 51,446 children were registered between 2003 and 2019, with 30,441 being excluded from our analysis due to registration after 6 weeks of age, lack of follow-up information, death by 6 weeks of age, missing baseline information, or emigration during follow-up (see flowchart in Figure S1 in Multimedia Appendix 1). The study sample included 21,005 children, which was weighted to an analytical sample of 27,998 children using IPCW to account for nonrandom emigration. The hypothesis-generating and temporal validation cohorts were based on weighted samples of 19,311 and 8687 children, respectively. The weights were not extreme (Multimedia Appendix 2). The mortality risk during the follow-up period (from 6 weeks to 3 years of age) was 5.2% (95% CI 4.8%-5.6%) in the hypothesis-generating cohort and 2.9% (95% CI 2.5%-3.3%) in the temporal validation cohort.

### Spatial Analysis

**Figure 2:**
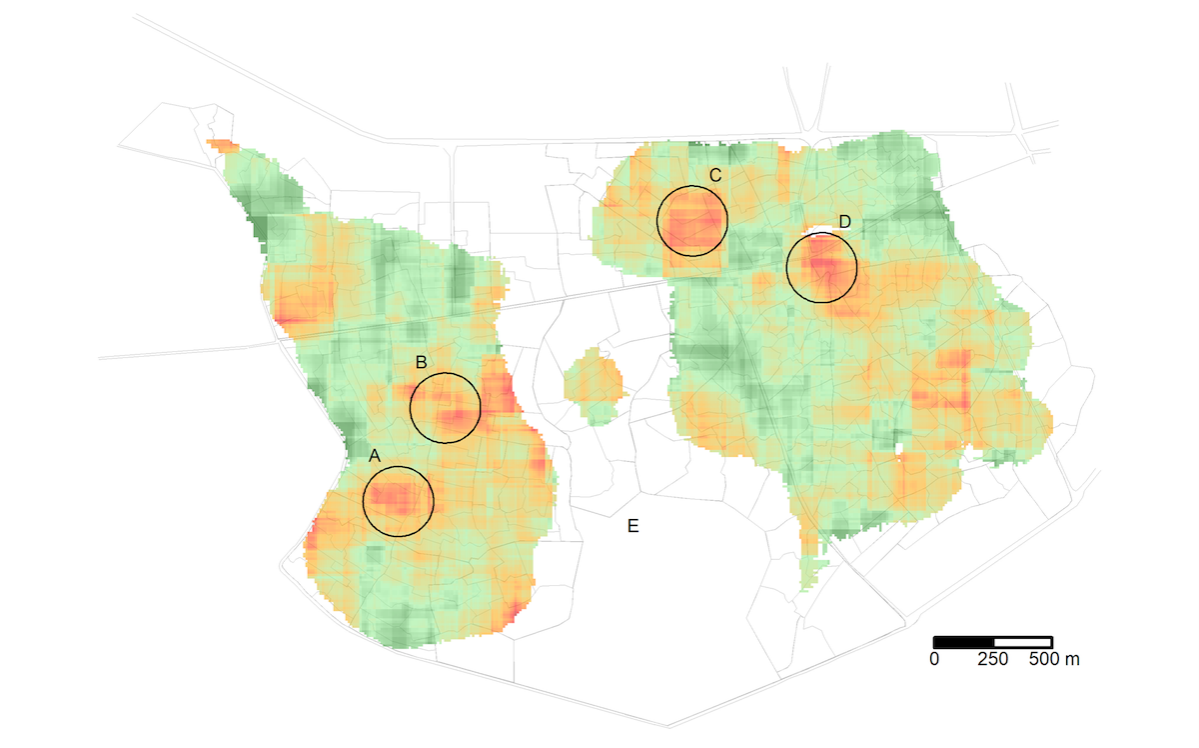
Spatial analysis mortality risk among the hypothesis-generating cohort adjusting for a linear effect of calendar time. Mortality risk (deaths per 100 children) in 250 m × 250 m squares by a resolution of 10 m for the birth years 2003-2011. Results are only shown if at least 100 children were under observation. A, B, C, and D indicate areas with a high child mortality risk. E is an uninhabited area, which is flooded during the rainy season.

We explored the results from the spatial analysis of the hypothesis-generating cohort, which gave rise to defining 4 areas; A, B, C, and D where the child mortality rate was considerably high. We marked these areas with circles on top of the spatial results in Figure 2. By comparing children living in the residential areas marked by circles A, B, C, and D (constituting between 1% [n=253] and 3% [n=533] of children) to those living outside these areas (Figure 2), the aMRD was 4.5% (95% CI –0.6% to 9.6%), 1.9% (95% CI –1.2% to 5.0%), 3.3% (95% CI –0.3% to 6.9%), and 4.0% (95% CI 0.1%-8.0%), respectively. When the 4 suggested high-risk residential areas were assessed in the temporal validation cohort, only area D still tended to exhibit higher mortality though the estimate was associated with more uncertainty (aMRD of 2.0%, 95% CI –2.8% to 6.7%) (Table 1 and Figure S4 in Multimedia Appendix 1). The combined estimate for both cohorts for area D was an aMRD of 3.4% (95% CI 0.3%-6.5%). If causal, the excess risk translates to a PAF of 1.1% of all deaths.

**Table 1:** Summary and validations of findings relevant for hypotheses-generations for targeted interventions.

			Hypothesis-generating cohort									Temporal validation cohort									aMRD^a^ visualization: full=hypothesis-generating cohort, dashed=temporal validation cohort			Test for similar aMRD, *P* value; TMLE^b^ estimate of both cohorts, 95% CI; population attributable fraction based on the estimate of both cohorts, %
			Prevalence (n=19,311), n (%)		Crude mortality risks (95% CI) within subgroup versus the rest		Synergistic associations, 95% CI^c^		TMLE aMRD (deaths per 100 children) in subgroup versus the rest, 95% CI^d^		Prevalence (n=8687), n (%)			Crude mortality risks (95% CI) within subgroup versus the rest		Synergistic associations, 95% CI^c^		TMLE aMRD (deaths per 100 children) in subgroup versus the rest, 95% CI^d^						
**Spatial analysis**																								
	A	1.3 (253)		9.7 (6.4 to 12.9) vs 5.1 (4.8 to 5.5)		N/A^e^		4.5 (–0.6 to 9.6)^f^		1.2 (106)			2.4 (–1.3 to 6.1) vs 9 (2.5 to 3.3)		N/A		–0.6 (–3.9 to 2.8)^f^		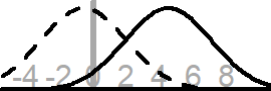			.10; 3.0 (–0.7 to 6.6)^f^; 0.8		
	B	2.0 (381)		6.9 (4.3 to 9.6) vs 5.2 (4.8 to 5.5)		N/A		1.9 (–1.2 to 5.0)^f^		1.5 (127)			1.0 (–2.4 to 4.4) vs 3.0 (2.6 to 3.4)		N/A		–2.2 (–4.0 to –0.4)^f^		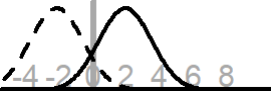			.02; 0.9 (–1.4 to 3.2)^f^; 0.4		
	C	2.8 (533)		8.4 (6.1 to 10.6) vs 5.1 (4.7 to 5.5)		N/A		3.3 (–0.3 to 6.9)^f^		2.7 (232)			2.3 (–0.2 to 4.8) vs 2.9 (2.5 to 3.4)		N/A		–0.7 (–2.9 to 1.6)^f^		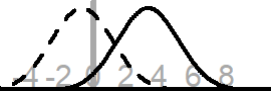			.07; 2.1 (–0.6 to 4.7)^f^; 1.2		
	D	1.6 (314)		9.0 (6.1 to 12.0) vs 5.1 (4.8 to 5.5)		N/A		4.0 (0.1 to 8.0)^f,g^		1.2 (104)			4.7 (1.0 to 8.5) vs 2.9 (2.5 to 3.3)		N/A		2.0 (–2.8 to 6.7)^f,g^		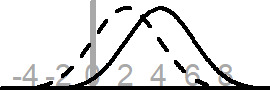			.51; 3.4 (0.3 to 6.5)^f^; 1.1		
**Single risk factors**																								
	Below 7 years of maternal education	60.8 (11,739)		5.9 (5.5 to 6.4) vs 4.1 (3.5 to 4.6)		N/A		1.6 (0.8 to 2.4)^h^		48.6 (4218)			3.6 (3.0 to 4.2) vs 2.3 (1.7 to 2.8)		N/A		1.3 (0.5 to 2.1)^h^		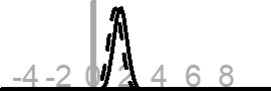			.60; 1.5 (0.9 to 2.1)^h^; 19.4		
	More children in the household younger than 3 years)	83.1 (16,054)		5.5 (5.1 to 5.9) vs 3.8 (2.9 to 4.6)		N/A		1.6 (0.6 to 2.6)^h^		79.1 (6874)			3.3 (2.8 to 3.7) vs 1.6 (0.7 to 2.5)		N/A		1.8 (0.9 to 2.6)^h^		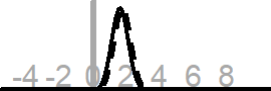			.78; 1.6 (0.9 to 2.4)^h^; 30.0		
	Mothers not under HDSS^i^ surveillance	3.6 (690)		19.1 (17.1 to 21.0) vs 4.7 (4.3 to 5.0)		N/A		16.7 (9.8 to 23.6)^h^		0.1 (11)			Very few children		Very few children		Very few children		N/A			N/A		
	No prenatal consultations	4.3 (834)		9.5 (7.7 to 11.3) vs 5.0 (4.6 to 5.4)		N/A		6.4 (2.5 to 10.2)^g,j^		3.4 (297)			6.0 (3.8 to 8.2) vs 2.8 (2.4 to 3.2)		N/A		2.8 (–0.9 to 6.6)^g,j^		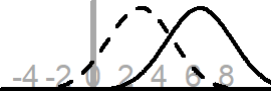			.20; 5.8 (2.6 to 8.9)^j^; 5.1		
**Multifactorial risk groups**																								
	Being a twin and born in the rainy season	1.6 (315)		12.4 (9.5 to 15.3) vs 5.1 (4.7 to 5.4)		6.8 (2.7 to 10.9)		7.0 (2.0 to 11.9)^j^		1.9 (162)			5.8 (2.8 to 8.8) vs 2.9 (2.5 to 3.3)		–5.3 (–9.7 to –0.9)		1.3 (–3.0 to 5.6)^j^		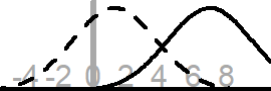			.09; 6.0 (2.3 to 9.8)^j^; 2.3		
	Children of polygamous families born in the dry season	9.2 (1770)		7.2 (6.0 to 8.4) vs 5.0 (4.6 to 5.4)		1.9 (0.0 to 3.8)		1.8 (0.2 to 3.3)^g,j^		8.3 (720)			5.7 (4.3 to 7.2) vs 2.7 (2.3 to 3.1)		2.0 (–0.2 to 4.1)		1.7 (–0.4 to 3.9)^g,j^		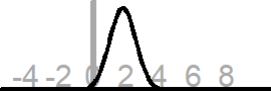			.97; 1.7 (0.4 to 2.9)^j^; 3.3		
	No prenatal consultations, boys, and other ethnicity	0.3 (61)		Very few children		Very few children		Very few children		0.2 (20)			Very few children		Very few children		Very few children		N/A			N/A		
	Mothers not under HDSS surveillance, Fula, or Mandinga ethnicity, born in the dry season	0.6 (124)		23.2 (18.6 to 27.9) vs 5.1 (4.7 to 5.4)		5.5 (0.5 to 10.5)		1.0 (–16.1 to 18.1)^j^		0.0 (0)			Very few children		Very few children		Very few children		N/A			N/A		

^a^aMRD: adjusted mortality risk difference.

^b^TMLE: targeted maximum likelihood estimation.

^c^The additional risk above the linear effect of the single factors is presented (ie, the parameter for the subgroup parameter while adjusting for each of the variables used to create the subgroup definition). Also adjusted for calendar time but no potential confounders.

^d^The additional risk-adjusted using targeted maximum likelihood estimation with using linear models.

^e^N/A: not applicable.

^f^Adjustment for calendar time.

^g^These findings were considered consistent across the cohorts by the authors.

^h^Adjustment for calendar time and environmental factors.

^i^HDSS: Health and Demographic Surveillance Systems.

^j^Adjustment for calendar time and environmental and household factors.

### Single Risk Factors

In the hypothesis-generating cohort, children of mothers with less education than 7 years compared to those with 7 years or more of education were common (n=11,739, 60.8%) and had an aMRD of 1.6% (95% CI 0.9%-2.4%; Table 2). Crowding (ie, having multiple children in the household under 3 years of age) was common (n=16,054, 83%) and associated with an aMRD of 1.6% (95% CI 0.6%-2.6%) compared to being the sole child (Table 2). Having functioning electricity, a television, and a toilet inside the house indicates higher wealth, which was associated with lower child mortality. Across both cohorts with very similar estimates, both less maternal schooling and more crowding were associated with an aMRD of approximately 1.5% (Table 1, the column “aMRD visualizations” shows virtually the same estimates). If causal 19.4% of all deaths could be attributed to low-maternal education and 30.0% to crowding.

The most pronounced environmental factor was living within 50 m of a major road, associated with an aMRD of 2.1% (95% CI 0.6%-3.6%) compared with children living further away.

Children of mothers lost to follow-up were at a marked increased mortality risk. Still, they constituted a very small number of children in the temporal validation cohort (additional explanation in Table S1 in Multimedia Appendix 1).

Being a twin was consistently associated with higher mortality, with an aMRD of 4.3% (95% CI 1.0%-7.7%) and 3.0% (95% CI –0.5% to 6.5%) in the hypothesis-generating and temporal validation cohorts, respectively (Table 1).

No prenatal consultation was recorded for 4% (n=834) of the children in the hypothesis-generating cohort and was associated with an aMRD of 6.4% (95% CI 2.5%-10.2%; Table 1). In the temporal validation data set, this was associated with an aMRD of 2.8% (95% CI –0.9% to 6.6%).

**Table 2:** The association between single risk factors and child mortality below 3 years of age. Hypothesis-generating cohort (Bandim Health Project Health and Demographic Surveillance Systems data from the birth years from 2003 to 2011).

Baseline information and category			Prevalence of children (n=19,311), n (%)	Deaths per 100 children, n	Unadjusted mortality risk difference (95% CI; additional deaths per 100 children)	Adjusted mortality risk difference^a^ (95% CI; additional deaths per 100 children)
**Environmental**						
	**Vegetation near the household at birth**					
		The 90% least vegetation	17,890 (93^b^)	5.2	0 (ref^c^)	0 (ref)
		The top 10% vegetation	1421 (7^b^)	5.1	–0.1 (–1.5 to 1.2)	–0.1 (–1.5 to 1.3)
	**Distance to a major road**					
		Within 50 m	1178 (6)	3.2	0 (ref)	0 (ref)
		More than 50 m	18,133 (94)	5.3	2.1 (0.6 to 3.6)	2.1 (0.6 to 3.6)
	**Population density 500 m × 500 m**					
		90% lowest density	17,659 (91^b^)	5.2	0 (ref)	0 (ref)
		10% highest density	1652 (9^b^)	5.6	0.5 (–0.8 to 1.8)	0.4 (–0.9 to 1.7)
	**Vaccination against tuberculosis in the local area**					
		Below 80%	551 (3)	4.6	0 (ref)	0 (ref)
		80% or above	18,760 (97)	5.2	0.6 (–1.6 to 2.8)	0.4 (–1.8 to 2.6)
	**Distance to a health center**					
		≤1 km	17,849 (92)	5.1	0 (ref)	0 (ref)
		>1 km	1462 (8)	6.0	0.9 (–0.5 to 2.3)	1.0 (–0.4 to 2.3)
**Household**						
	**Mother lost to follow-up**					
		Yes	18,621 (96)	4.7	0 (ref)	0 (ref)
		No	690 (4)	19.1	14.4 (12.5 to 16.3)	14.4 (12.5 to 16.4)
	**Whether the mother lives with the father**					
		Yes	12,255 (63)	4.8	0 (ref)	0 (ref)
		No	7056 (37)	5.8	1.0 (0.2 to 1.7)	1.0 (0.3 to 1.8)
	**Roof**					
		Zinc	18,581 (96)	5.1	0 (ref)	0 (ref)
		Other	730 (4)	6.3	1.1 (–0.8 to 3.0)	0.9 (–1.0 to 2.8)
	**Electricity**					
		Yes	5995 (31)	4.5	0 (ref)	0 (ref)
		No	13,317 (69)	5.5	1.0 (0.2 to 1.7)	0.9 (0.1 to 1.7)
	**Television**					
		Yes	6338 (33)	4.4	0 (ref)	0 (ref)
		No	12,973 (67)	5.6	1.2 (0.5 to 2.0)	1.0 (0.3 to 1.8)
	**Toilet**					
		Inside the house	2916 (15)	4.3	0 (ref)	0 (ref)
		Other	16,395 (85)	5.3	1.0 (0.0 to 2.0)	0.8 (–0.2 to 1.9)
	**Maternal schooling (years)**					
		≥7	7572 (39)	4.1	0 (ref)	0 (ref)
		<7	11,739 (61)	5.9	1.9 (1.1 to 2.6)	1.6 (0.9 to 2.4)
	**Polygamous families**					
		No	15,803 (82)	5.0	0 (ref)	0 (ref)
		Yes	3508 (18)	6.2	1.2 (0.3 to 2.1)	1.1 (0.2 to 2.1)
	**Mother works outside of the home**					
		Yes	555 (3)	4.3	0 (ref)	0 (ref)
		No	18,756 (97)	5.2	0.9 (–1.3 to 3.1)	0.9 (–1.2 to 3.1)
	**Ethnicity**					
		Balanta	1637 (8)	4.5	0 (ref)	0 (ref)
		Fula or Madinga	5359 (28)	4.9	0.3 (–1.1 to 1.8)	0.4 (–1.0 to 1.8)
		Manjaco or Mancanha	3758 (19)	5.3	0.7 (–0.8 to 2.2)	0.8 (–0.7 to 2.3)
		Pepel	3154 (16)	5.3	0.8 (–0.8 to 2.3)	0.9 (–0.6 to 2.4)
		Other	5404 (28)	5.6	1.0 (–0.4 to 2.4)	1.1 (–0.4 to 2.5)
	**Other children below 3 years in the household**					
		No	3257 (17)	3.8	0 (ref)	0 (ref)
		Yes	16,054 (83)	5.5	1.7 (0.8 to 2.7)	1.6 (0.7 to 2.6)
**Information related to delivery**						
	**Sex**					
		Boy	9880 (51)	5.3	0 (ref)	N/A^d^
		Girl	9431 (49)	5.1	–0.3 (–1.0 to 0.5)	N/A
	**Twin**					
		No	18,696 (97)	5.1	0 (ref)	0 (ref)
		Yes	615 (3)	9.4	4.3 (2.2 to 6.4)	4.3 (2.2 to 6.3)
	**Birth season**					
		Dry	9836 (51)	5.4	0 (ref)	N/A
		Rainy	9475 (49)	4.9	–0.5 (–1.2 to 0.2)	N/A
	**Place of birth**					
		Hospital or health center	13,786 (71)	5.0	0 (ref)	0 (ref)
		At home	5525 (29)	5.6	0.6 (–0.2 to 1.4)	0.1 (–0.8 to 0.9)
	**Maternal age (years)**					
		>25	9780 (51)	4.9	0 (ref)	0 (ref)
		≤25	9531 (49)	5.5	0.6 (–0.1 to 1.4)	0.4 (–0.4 to 1.2)
	**Firstborn**					
		Not firstborn	13,389 (69)	5.0	0 (ref)	0 (ref)
		Firstborn	5922 (31)	5.6	0.5 (–0.2 to 1.3)	0.6 (–0.3 to 1.4)
	**Born by cesarean section**					
		Yes	950 (5)	4.0	0 (ref)	0 (ref)
		No	18,361 (95)	5.3	1.2 (–0.5 to 2.9)	1.2 (–0.5 to 2.9)
	**Prenatal consultations**					
		Yes	18,478 (96)	5.0	0 (ref)	0 (ref)
		No	834 (4)	9.5	4.5 (2.7 to 6.3)	3.9 (2.1 to 5.7)

^a^For the environmental category, adjusted risk differences are adjusted for a calendar effect, risk differences in the household category are additionally adjusted for the environmental variables, and risk differences in the information related to the delivery category are further adjusted for the household variables.

^b^The prevalence differs from 10% to 90% because the cutoff is based on data from both cohorts (2003-2016).

^c^Reference group.

^d^N/A: not applicable, since the effect of sex and birth season is not expected to be confounded and thus does not need adjustment.

### Multifactorial Risk Groups

**Figure 3:**
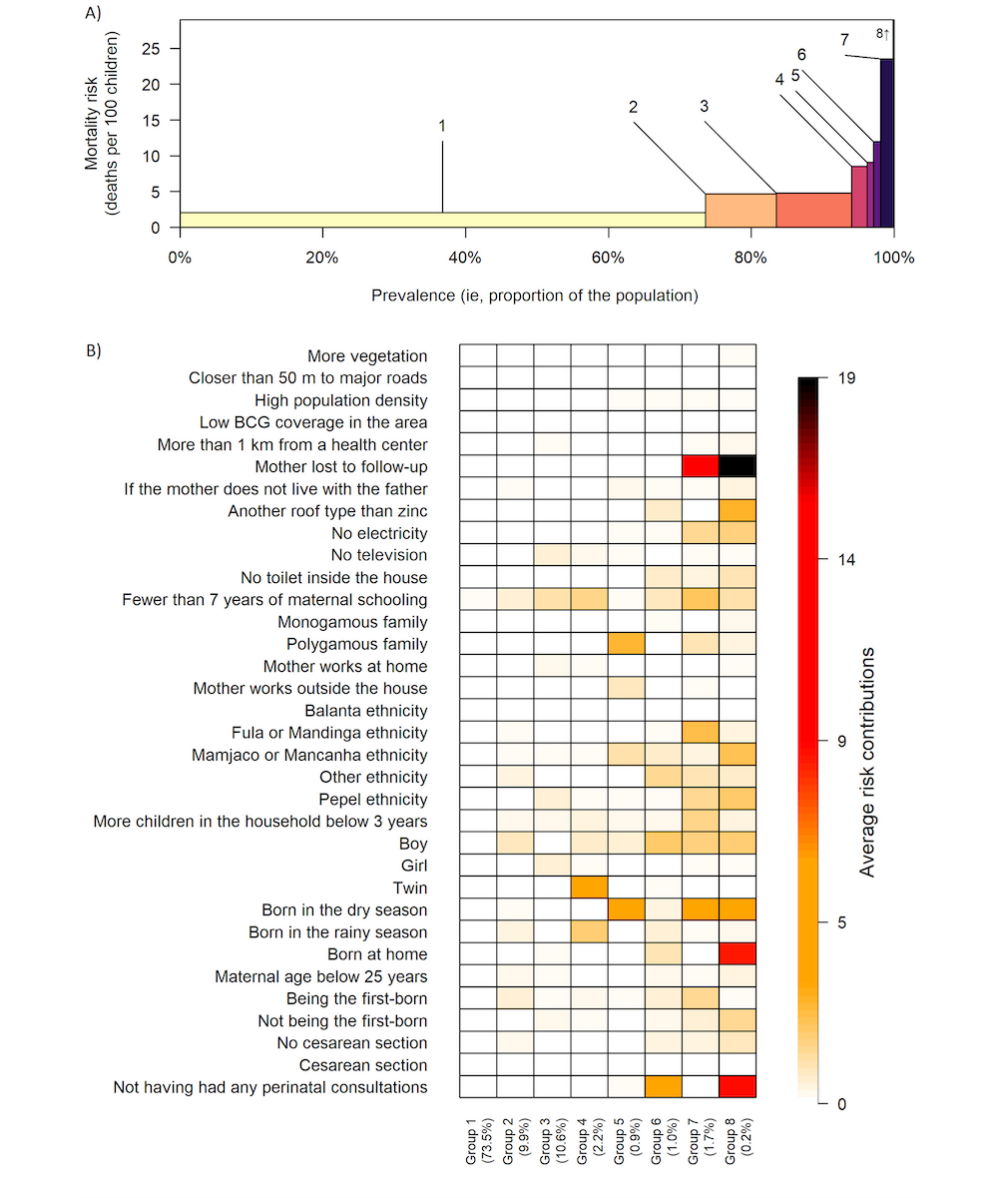
Multifactorial risk groups results from the Causes of Outcome Learning approach. (A) The prevalence and mean risk by the identified subgroups based on the Causes of Outcome Learning approach. Group 8 had a higher mortality risk than could be shown in the plot. (B) The mean risk contributions by each subgroup are visualized, where the color white indicates no risk contribution and dark red indicates the highest risk contribution. We identify 4 subgroups with a prevalence above 0.5% (n=97): (1) twins born in the rainy season (group 4); (2) children of polygamous families born in the dry (group 5); (3) no consultations, boys, and other ethnicity (group 6); (4) mothers not under Health and Demographic Surveillance Systems surveillance, Fula, or Mandinga ethnicity, born in the dry season (group 7). For more details, see Multimedia Appendix 3. BCG: Bacillus Calmette-Guérin vaccination.

In the hypothesis-generating cohort, twins born in the rainy season had higher mortality risk compared with those born in the dry season (aMRD of 7.0%, 95% CI 2.0%-11.9%; Figure 3, group 4, and Table 1), but this association was not found in the temporal validation data (aMRD 1.3%, 95% CI –3.0% to 5.6%; Table 1). Children of polygamous families born in the dry season had an aMRD of 1.8% (95% CI 0.2%-3.3%) compared to all other children (Figure 3, group 5, and Table 1). This subgroup constituted 9% (n=1770) of the children in the hypothesis-generating cohort, and the finding was consistent in the temporal validation cohort (an aMRD of 1.7%, 95% CI –0.4% to 3.9%, covering 8% [n=720] of all children; Table 1, see the column with aMRD visualization for consistency). If these associations are causal, 3.3% of all deaths could be attributed to this combination. A supplementary analysis of both cohorts (birth years 2003-2016) was conducted to understand the phenomenon better. The results suggested that (1) the finding was not artificially introduced by the IPCW approach; (2) the increased risk was highest in the first half-year of follow-up (6 weeks to 7 months of age) but continued throughout the entire follow-up period (up to 3 years of age); (3) the association varied across birth years without any trend; (4) the association was strongest among the Manjaco and Mancanha ethnic groups; (5) the association was most pronounced in the eastern part of the HDSS area; and (6) the association was not confounded by crowding, but was driven by the strata of children living in a household with other children under 3 years of age (Multimedia Appendix 4).

## Discussion

### Principal Findings

In this study, we aimed to discover subgroups of children with high mortality in urban Guinea-Bissau. We used complementary analyses and split the data into a hypothesis-generating cohort and a temporal validation cohort. Of children with high mortality who may be targeted for interventions, we identified (1) a residential area (area D), (2) children of mothers who did not attend prenatal consultations, and (3) children born in polygamous families during the dry season had excess mortality risk throughout the study period. Of population-wide findings, maternal education and household crowding were important factors.

### Limitations

Excluding children without complete information (Figure S1 in Multimedia Appendix 1), and conditioning on children being alive at 6 weeks of age [19] may have limited the generalizability of our findings. The HDSS is a valuable source of information but is focused on specific key health indicators as data collection in Guinea-Bissau is resource-demanding. Thus, we lacked information about other relevant baseline characteristics such as vector-borne diseases, health care and systems, weather, pollution, water and sanitation, community relations (social capital), and household and macroeconomic conditions. We did not include factors that varied during follow-up, such as season, vaccinations, vitamins, and other health campaigns. This may be important as child mortality is considerably higher in the rainy season [19,20]. Live and nonlive vaccines have been shown to affect child mortality more generally than their effects on the targeted diseases explained [21].

We acknowledge that data gathered from the real world may have some natural limitations in terms of completeness and accuracy, which could potentially affect the reliability of the identified risk factors. Efforts to account for missing or incomplete data were made where feasible. Furthermore, as this study concentrates on urban Guinea-Bissau, its findings may not readily apply to different socioeconomic and cultural contexts. Further research in varied settings is necessary to validate and understand the transferability of these factors.

To acknowledge the challenges in establishing causality, we integrated multiple methods and used an inductive-deductive research methodology (ie, take the learnings from the hypothesis-generating cohort to be tested in the temporal validation cohort) [22]. This approach guided us to propose future research directions to validate and understand the mechanisms driving the observed phenomena. While total effects can be diluted in the Causes of Outcome Learning approach, particularly when including individual and birth-related factors in the model (Figure 3), our methodology is strengthened by using a causal structure (Table 1) for adjustments and the TMLE approach. This enhances the validity of our findings and contributes toward a more robust inference of causality by better adjustment and more robust model specification [17]. It could be explored if other novel machine learning methods could supplement the findings [23].

### Interpretation

The temporal split allowed us to investigate consistency across 2 time periods. While a lack of consistency can be due to chance, it may also reflect changes in the causal structure over time. We found that children of mothers not under HDSS surveillance were strongly associated with child mortality in the hypothesis-generating cohort. However, in the temporal validation cohort, close to no children had mothers who were not under HDSS surveillance. Changes in data collection methods might explain this discrepancy; after 2013, mothers of children in new families were registered by the same data collector as the child, whereas before 2013, the mother’s registration was handled by a separate team. The increased risk among twins born in the rainy season in the hypothesis-generating cohort may have occurred by chance. Still, it could also indicate better health care for high-risk children in the validation cohort.

### Local Environmental Factors

Previous spatial studies have shown large differences in disparities within and between countries [24,25], and temporal persistence at local levels [26]. The population movement in Bissau may have made it more difficult to identify high-risk residential areas. Area D contains a busy market called Caracol which is known for traditional medicine and care. High population density and possibly high infectious load, may offer 1 explanation for the high risk in residential area D. The proportion of mothers with less than 7 years of schooling was similar in area D as outside of it (Figure S5 in Multimedia Appendix 1).

To further understand and address the high mortality rate in this residential area, several future studies could be conducted, such as (1) qualitative study following families in this area may add insight and create new hypotheses; (2) network analysis to reveal contact patterns and exposed jobs most relevant in this area; and (3) spatial analysis of distance to specific proximate places (eg, places for traditional medicine and care), infrastructures (eg, wells), or potentially hazardous areas (eg, waste collection areas).

### Lack of Prenatal Consultations

Prenatal consultations are designed to prevent early child mortality and may directly affect maternal behavior. The association between lack of a prenatal consultation and mortality is reflected in other studies [27], but we cannot exclude that some of the association was confounded by social and economic factors, as well as health care-seeking behavior. This may be especially important as we are considering postneonatal mortality. Various mechanisms may contribute to postneonatal mortality, such as out-of-pocket fees associated with increased child mortality in sub-Saharan Africa [28]. In Guinea-Bissau, the expansion of free antenatal care was, however, not associated with reduced perinatal mortality [29], and thus some of the observed associations of prenatal consultations may reflect confounding.

To further understand and address the lack of prenatal consultations and its impact on child mortality, a number of future studies could be conducted, such as (1) studies examining various characteristics of mothers not participating in prenatal consultation and their outcomes to understand further how this subgroup is associated with mortality and morbidity, (2) assess the effectiveness of interventions such as active home visits with prenatal consultations in reducing child mortality, and (3) explore if health care decisions during prenatal consultations can be assisted by artificial intelligence–based assessment systems [30].

### Family Type and Birth Season

Connecting children of polygamous families born in the dry season, an HDSS-based study from the Gambia identified that children born in the harvest season (January-June, approximately equivalent to the dry season in Guinea-Bissau which is December-May) were at increased postneonatal mortality risk [31] and a study from Ghana found that children from polygamous families had higher child mortality than those of monogamous families [32]. We could not identify other studies assessing the combination of family type and birth season. Within our study, we further observed that the finding was not indicated to be confounded by crowding, though residual confounding may persist. However, we found that the pattern was only present for children living in households with other children under 3 years of age. Some mothers travel to rural villages to harvest cashew nuts in the late dry season and return in the rainy season. One explanation may lie in the divided attention between labor in the cashew plantations and care for other children (potentially in a different environment). With reduced or limited breastfeeding during the cashew harvest, children may lose maternal antibodies and thus become more susceptible to infections. How these mechanisms interact with family structure is still to be understood.

To further understand and address the association between birth season and family type on child mortality, several future studies could be conducted, such as (1) investigation into accidents as causes of death may reveal if the combination of shared child attendance and busy months in relation to the harvest increases the risk of domestic accidents, (2) interviews with these families may give insight into the observed phenomenon, and (3) triangulating the findings with other health-related behaviors such as vaccination uptake may help uncover mechanisms.

### Resource Prioritization

As repeatedly described in the literature, social and economic factors affecting a wide part of the population strongly predict mortality [33]. In our data, social and economic factors may account for 20%-30% of all deaths in children aged 6 weeks to 3 years. In contrast, the 3 subgroups of children with high mortality identified in our study may represent a smaller fraction of the overall mortality burden (less than 5%), but they are characterized by significantly higher absolute mortality risks. This distinction raises important questions about the feasibility and potential impact of targeted interventions for these subgroups as compared to more widespread, universal public health strategies. While recognizing the challenges in reaching these smaller subgroups, targeted interventions could be crucial in addressing their disproportionately high mortality risks. Therefore, it is imperative to consider both cost-effectivity and equity in designing these interventions, ensuring they complement broader public health measures to provide comprehensive and effective child health care.

### Demonstration of a Novel Approach for Targeted Public Health Research

This study not only provides insights into child mortality in urban Guinea-Bissau but also demonstrates the practical application of the Causes of Outcome Learning approach [14] on real-world data. Our findings illustrate how this approach effectively deciphers complex patterns and suggests potential synergistic causes in public health data, revealing phenomena that would be overlooked by traditional analytical methods. Future research should focus on identifying when the Causes of Outcome Learning approach is most effective and on refining the methodology to improve its accuracy and adaptability for a variety of public health research questions and study designs.

### Conclusions

Reaching the Sustainable Development Goal of reducing under-5 child mortality to below 1 in 25 children by 2030 will require a range of interventions. By using several different and complementary approaches, we were able to identify subgroups of children at a high mortality risk that would not be evident otherwise. These high-risk children live in a specific area near a marked area known for traditional medicine and care, have mothers who did not attend prenatal consultations, and were born in the dry season and in polygamous families. We have suggested several future studies that may help explore these hypotheses. Potential targeted interventions should be evaluated in comparison with the impact of population-wide structural interventions both from cost-effectivity aspects and equity aspects and tested under proper evaluation schemes [34] to reduce child mortality.

## Appendix

Multimedia Appendix 1 The variable operationalisation, flowchart, pairwise associations between basic HDSS information, temporal overview, geo-spatial patterns of the temporal validation data adjusting for a linear effect of calendar time, and geo-spatial patterns of the prevalence of mothers with fewer than 7 years of schooling.

Multimedia Appendix 2 The inverse probability of censoring weights.

Multimedia Appendix 3 The Causes of Outcome Learning method.

Multimedia Appendix 4 The analysis regarding the birth season and polygamous families.

## Abbreviations

aMRD: adjusted mortality risk differences
HDSS: Health and Demographic Surveillance Systems
IPCW: inverse probability of censoring weights
MRD: mortality risk difference
PAF: population attributable fraction
TMLE: targeted maximum likelihood estimation
