# Supplementary material for: Discovering Subgroups of Children With High Mortality in Urban Guinea-Bissau: Exploratory and Validation Cohort Study
Source: JMIR Public Health Surveill. 2024 Apr 9;10:e48060. doi: 10.2196/48060 (PMC11040440; doi:10.2196/48060)
Supplement: Multimedia Appendix 1 [file publichealth_v10i1e48060_app1.pdf]

Multimedia Appendix 1

This multimedia appendix covers the variable operationalisation, flowchart, pairwise associations between basic HDSS information, temporal overview, geo-spatial patterns of the temporal validation data adjusting for a linear effect of calendar time, and geo-spatial patterns of the prevalence of mothers with fewer than 7 years of schooling.

Table S1. Variable operationalisation

Baseline information		Description
Environmental	Vegetation near the household [10% with most vegetation vs 90% with the least vegetation]	We used the Normalized Difference Vegetation Index (NDVI) data for the same season and year as the child's birthday. Few seasons were missing, thus the 2003 rainy season used 2004 data. The 2008 rainy season used the 2007 data. The 2012 rainy season used the 2011 data. The 2008 and 2009 rainy seasons used 2007 data. The cut-off in vegetation was based on data from both cohorts (2003-2016).
	A major road near the household [within 50 metres vs the rest]	We used open street map data. The following roads were identified as having heavy traffic: Estrada de Bor, Chapa, Zona 7, Avenida dos Combatentes da Liberdade da Pátria.
	Population density [10% highest density vs the rest]	Estimated based on the data on mothers living in the area based on data from both cohorts (2003-2016).
	Low local BCG coverage [below 80% vs the rest]	The proportion of BCG (against tuberculosis) vaccinated children before 28 days of age in the study area, who were visited between 28 days and 1 year of age (not only the study population) by sub-area and birth cohort.
	Distant health centres [more than 1 km vs the rest]	Distance to one of the following health centres: Bandim, Belem, Cuntum, and Cuntum-Madina.
Household	Mother lost to follow-up [yes, no]	Whether the mother has not been met as part of the HDSS surveillance after the entry date when the child is 6 weeks of age. After 2013, mothers of children in new families were registered by the same data collector registering the child, before 2013 the registration of the mother was handled by a separate team.
	Whether the mother lives with the father [yes, no]	Question asked as part of registering the child (conducted at the first visit after the child is born).
	Roof type [zinc, other]	Question asked as part of registering the child (conducted at the first visit after the child is born).
	Electricity independently of it being operational [yes, no]	Question asked as part of registering the child (conducted at the first visit after the child is born).
	TV [yes, no]	Question asked as part of registering the child (conducted at the first visit after the child is born).
	Toilet [indoor toilet, other]	Question asked as part of registering the child (conducted at the first visit after the child is born).
	Maternal schooling [>= 7 years, < 7 years]	Question asked as part of registering the child (conducted at the first visit after the child is born).
	Family type [Polygamous family, monogamous families]	Question asked as part of registering the child (conducted at the first visit after the child is born).
	The mother works outside of the home [yes, no]	Question asked as part of registering the child (conducted at the first visit after the child is born).
	Ethnicity [Pepel, Balanta, Fula/Madinga, Manjaco/Mancanha, other]	Question asked as part of registering the child (conducted at the first visit after the child is born).
	More children in the household below 3 years [yes, no])	Across all children in the study area, a count for the number of children below 3 years living in the same household at the date when the included child entered the study at 6 weeks of age.
Information related to	Sex [boy, girl]	Question asked as part of registering the child (conducted at the first visit after the child is born).
	Twin [yes, no]	Question asked as part of registering the child (conducted at the first visit after the child is born).
	Birth season [dry, rainy]	Based on the birthday. The dry season is considered to be from December to May, and the rainy season is considered to be from June to November.
	Place of birth [at home, hospital/health centre]	Question asked as part of registering the child (conducted at the first visit after the child is born).

	Maternal age [<=25 years, >25 years]	Based on the mother's birthday.
	Birth number [first born, not first born]	Question asked as part of registering the child (conducted at the first visit after the child is born).
	Born by caesarean section [yes, no]	Question asked as part of registering the child (conducted at the first visit after the child is born).
	Prenatal consultations [yes, no]	Question asked as part of registering the child (conducted at the first visit after the child is born).

Figure S1. Flowchart

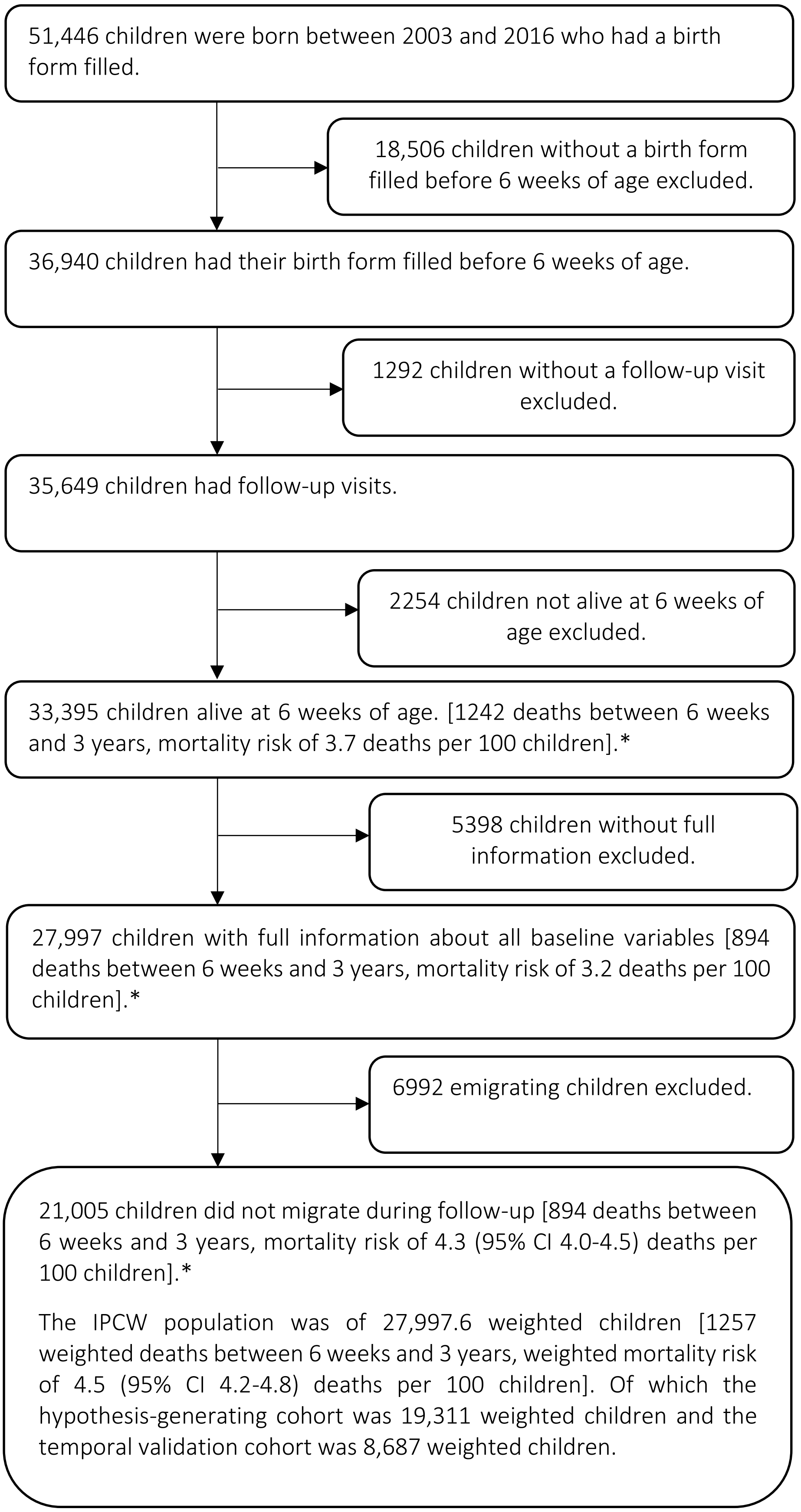

* The mortality risk is underestimated due because systematic censoring (i.e. emigration) is not yet accounted for.

Figure S2. Pairwise associations between basic HDSS information

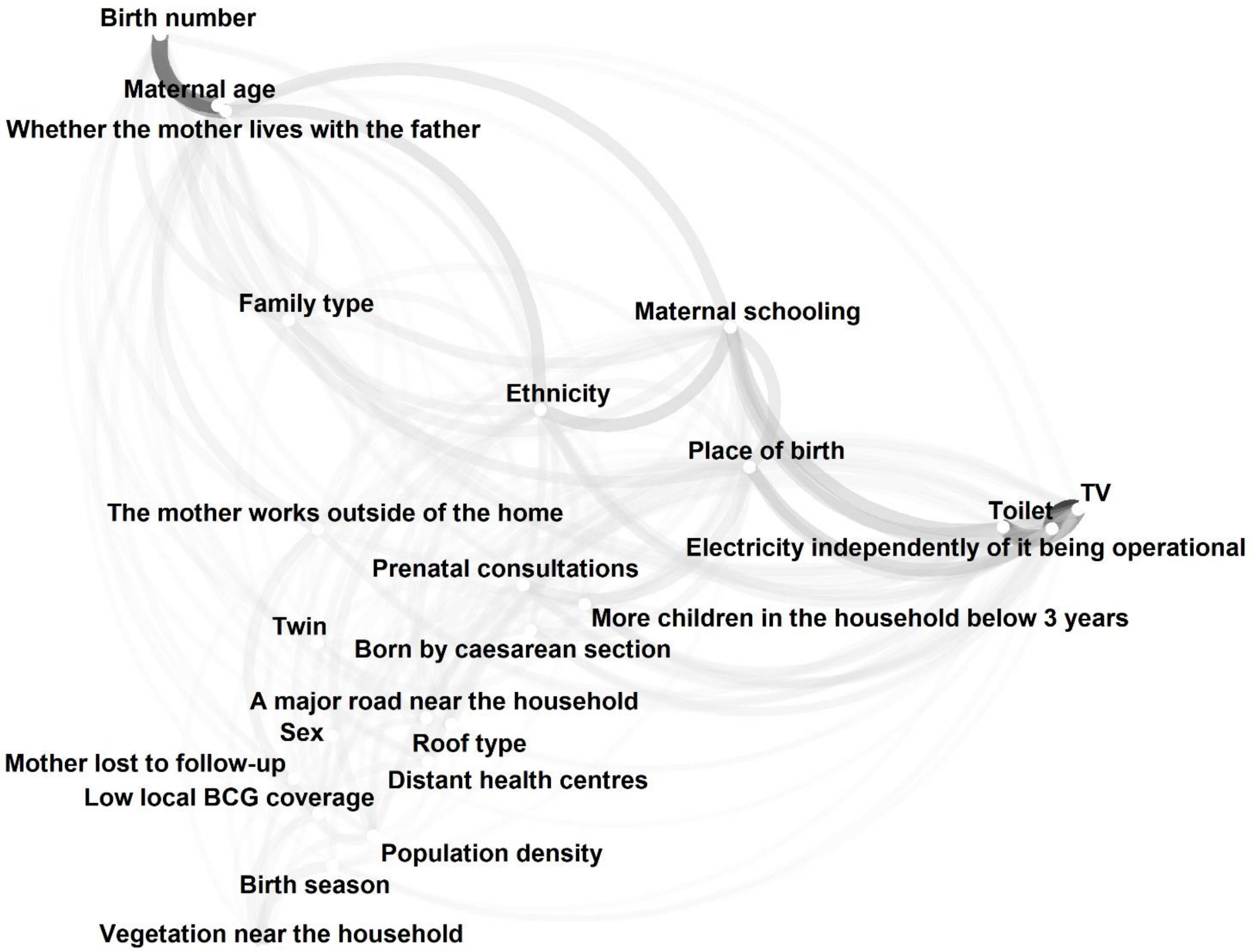

Increased width, contrast and proximity equally indicate stronger pairwise associations using Cramér's V. Economic possession indicators such as TV, toilet and connection to the electrical grid correlate. A mother's age and the birth number of the child correlate. Birth season and vegetation near the household at birth correlate.

Figure S3. Temporal overview

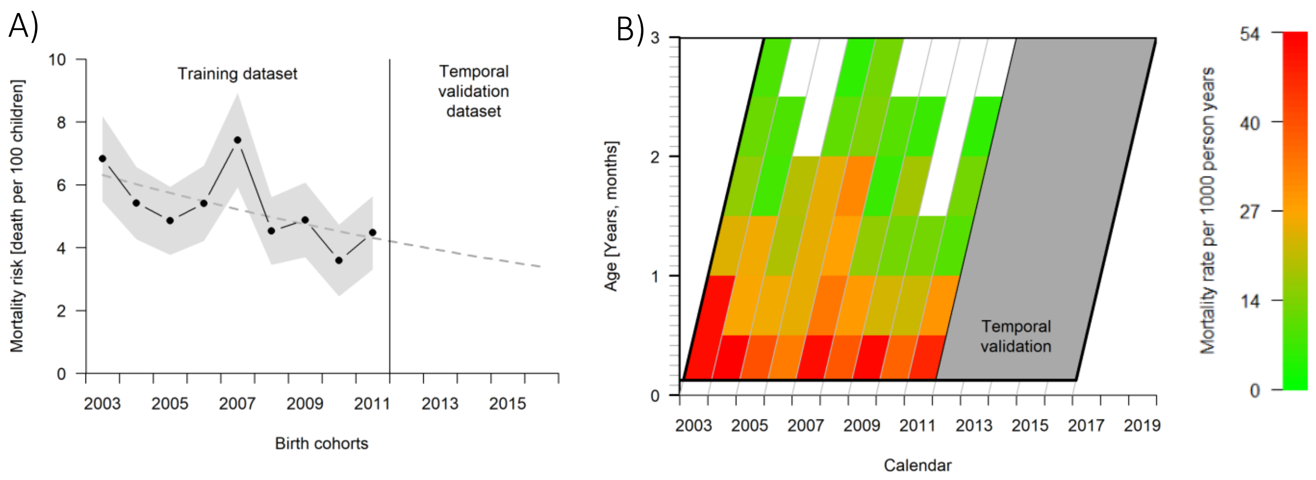

A) Deaths per 100 children reported for the follow-up period from 6 weeks to 3 years using a Kaplan-Meier estimator among children who survived until 6 weeks of age. The grey area shows the 95% confidence interval. All other analyses exclude children who are censored during the follow-up and use inverse probability of censoring weights to adjust for selection bias. B) Mortality rate by age and birth cohorts. The mortality rate is not shown for the white areas as they were based on fewer than 5 deaths. The birth cohort of 2007 appeared to have a higher child mortality rate compared with other years. The risk was elevated throughout their first 3 years of life and was especially driven by children born in the first quarter of 2007 (Supplementary analysis 1).

Figure S4. Geo-spatial patterns of the temporal validation data adjusting for a linear effect of calendar time

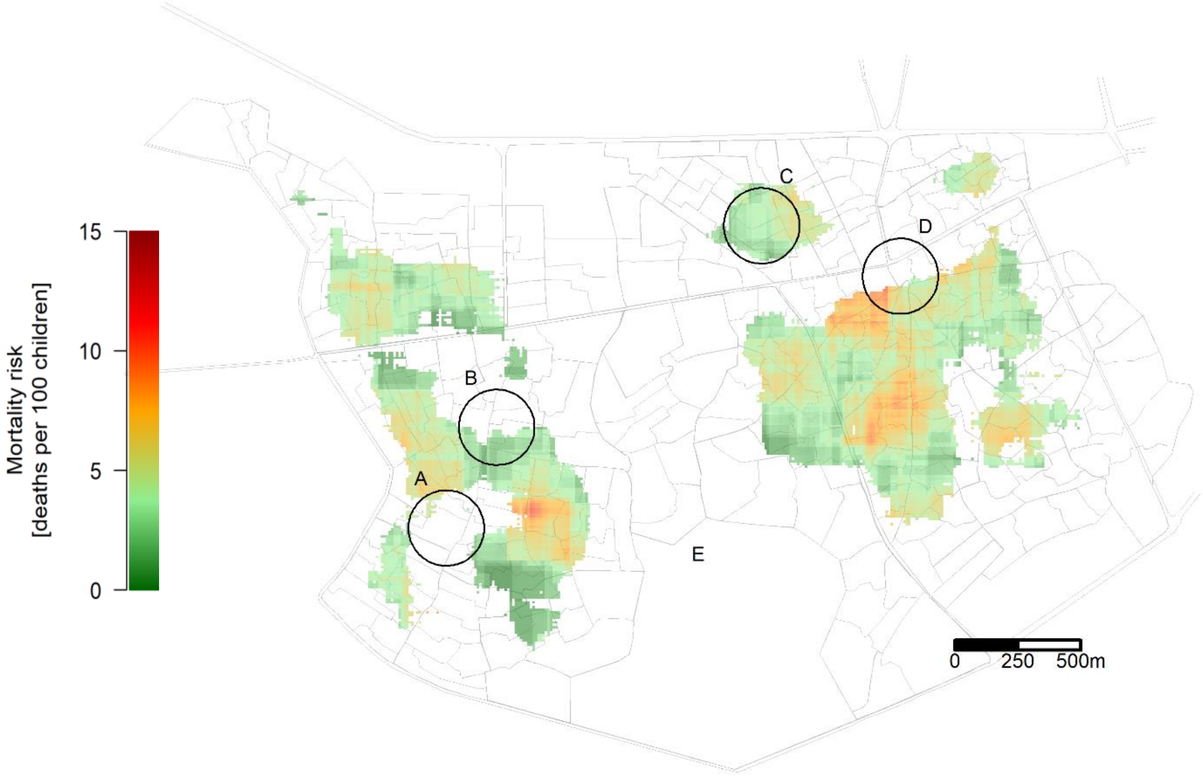

Mortality risk [deaths per 100 children] in 250 metres x 250 metres squares by a resolution of 10 metres for the birth cohorts 2012-2016. Results are only shown if at least 100 children were under observation. A, B, C, and D indicate areas that were associated with a high child mortality risk in the hypotheses generating cohort, however, only area D was associated with a high child mortality risk in the temporal validation dataset. E is an uninhabited area, which is flooded during the rainy season.

Figure S5. Geo-spatial patterns of the prevalence of mothers with fewer than 7 years of schooling

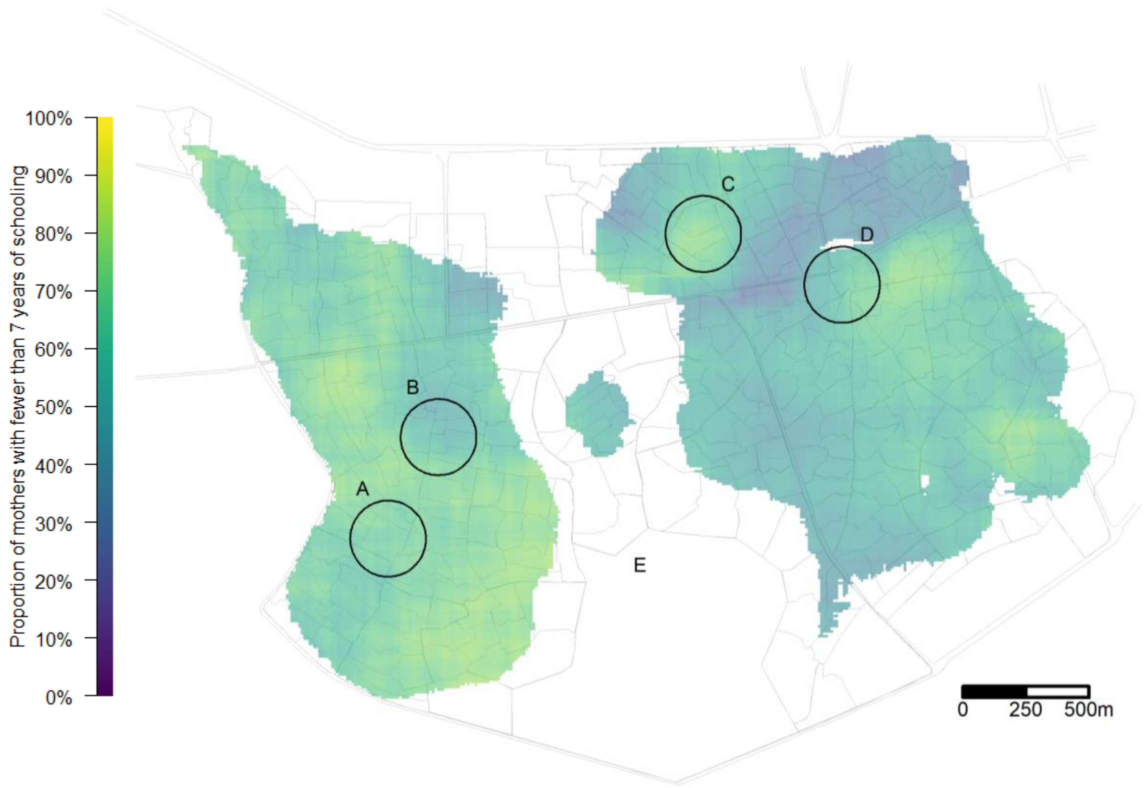

Prevalence of mothers with fewer than 7 years of schooling in 250 metres · 250 metres squares by a resolution of 10 metres for the birth cohorts 2003-2011 adjusting for a linear effect of calendar time. Results are only shown if at least 100 children were under observation. A, B, C, and D indicate areas with a higher child mortality risk. E is an uninhabited area, which is flooded during the rainy season.
