# Supplementary material for: Discovering Subgroups of Children With High Mortality in Urban Guinea-Bissau: Exploratory and Validation Cohort Study
Source: JMIR Public Health Surveill. 2024 Apr 9;10:e48060. doi: 10.2196/48060 (PMC11040440; doi:10.2196/48060)
Supplement: Multimedia Appendix 2 [file publichealth_v10i1e48060_app2.pdf]

Multimedia Appendix 2

This multimedia appendix covers the inverse probability of censoring weights.

Inverse probability of censoring weights (IPCW) can be used to adjust for selection bias.[9] IPCW are defined by:

$$IPCW = \frac{1}{P(C = 0|X)}$$

Where C = 0 denotes a child with full follow-up, and C = 1 denotes a child who emigrated during follow-up. X denotes the full set of all predictors of being censored. We estimated the propensity for not being censored, $P(C = 0|X)$, using logistic regression with all the basic information described in the method as well as birth year cohort modelled linearly. The weights were not extreme (<1% had a weight above 2). The weight distributions for the hypotheses-generating dataset were as below.

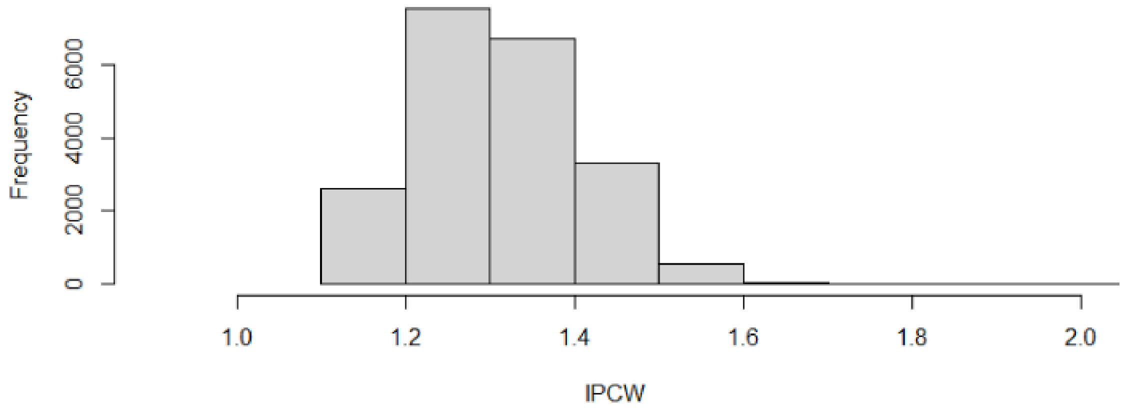

Due to rounded values, a study population that has been reweighted using IPCW may result only in approximately the same number of weighted individuals (e.g. we had 27,997 children of which 6,992 migrated, the IPCW population was 27,997.6 weighted children).
