# Supplementary material for: Discovering Subgroups of Children With High Mortality in Urban Guinea-Bissau: Exploratory and Validation Cohort Study
Source: JMIR Public Health Surveill. 2024 Apr 9;10:e48060. doi: 10.2196/48060 (PMC11040440; doi:10.2196/48060)

Multimedia Appendix 3

This multimedia appendix covers the Causes of Outcome Learning method.

The following baseline variables were assumed to have monotone effects increasing child mortality:

- More vegetation, closer than 50 metres to major roads, high population density, low BCG coverage in the area, more than 1 km from a health centre, mother lost to follow-up, if the mother does not live with the father, another roof type than zinc, no electricity, no tv, no toilet inside the house, fewer than 7 years of maternal schooling, more children in the household below 3 years, being a twin, born at home, maternal age below 25 years, and not having had any prenatal consultations.

The remaining baseline variables were allowed to have effects that were not necessarily monotonic by including the variables as binary / one-hot encoded in the monotonic neural network.

- Family type, working at home or outside, ethnicity, sex, birth season, birth order, and caesarean section.

We accounted for selection by inverse probability of censoring weights, and we adjusted for a calendar time effect by including the effect of calendar time as a linear effect on the outcome independently of the model (as a residual connection to the output layer in the neural network). A monotonic neural network with 30 activation functions was trained with a learning rate of 1e-4 and regularisation of the input parameters of 1e-4 until the performance had not improved for 500 epochs then with a learning rate of 1e-5 and then 1e-6. Below is shown the performance (A), baseline risk (B), and model (C). The linear effect of calendar effect was estimated to 2.6 fewer deaths per 100 children from 2003 to 2011. The area under the receiver operating characteristic curve (ROC AUC) in the hypothesis-generating cohort 0.64.

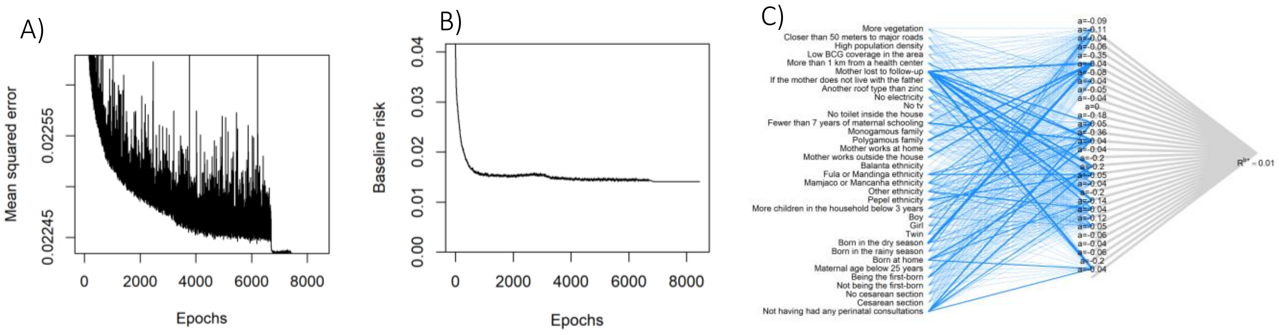

We created a tree visualisation of the distance matrix of the risk contributions using Manhattan distances (A), and calculated the within mean within subgroup risk contribution difference between all individuals conditioned of 1 to 20 subgroups clustered based on hierarchical clustering using the Ward method (B), and we chose 8 subgroups. Due to computational limitations, the hierarchical clustering was conducted without weights. The calibration curve indicated a good fit when comparing the predicted mortality risk by subgroup and their actual mortality risk. Group 8 was assigned a lower predicted risk than they actually have (C).

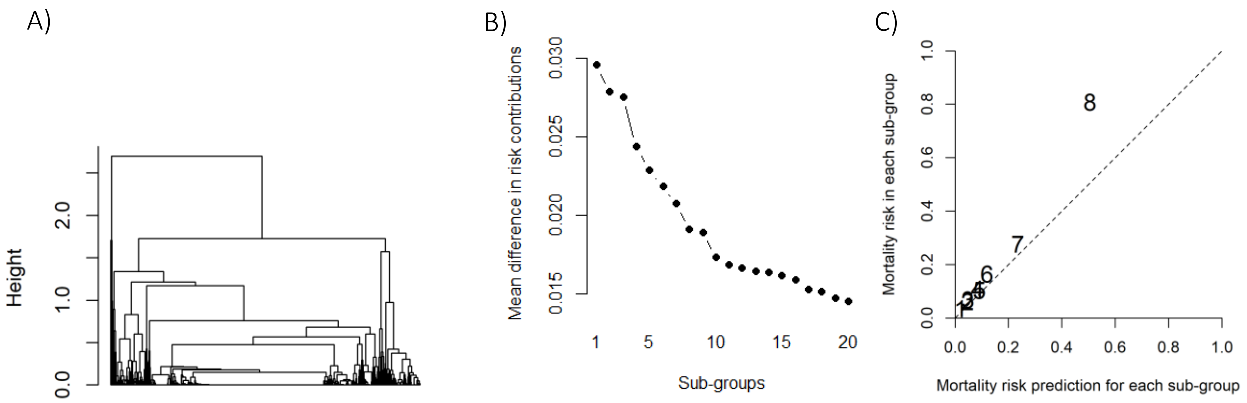

Sensitivity analyses allowing both directions of vegetation did not change the results of CoOL. We reran the Causes of Outcome Learning approach six times to assess consistency. It showed a relative consistency of the identified subgroups (below figures).

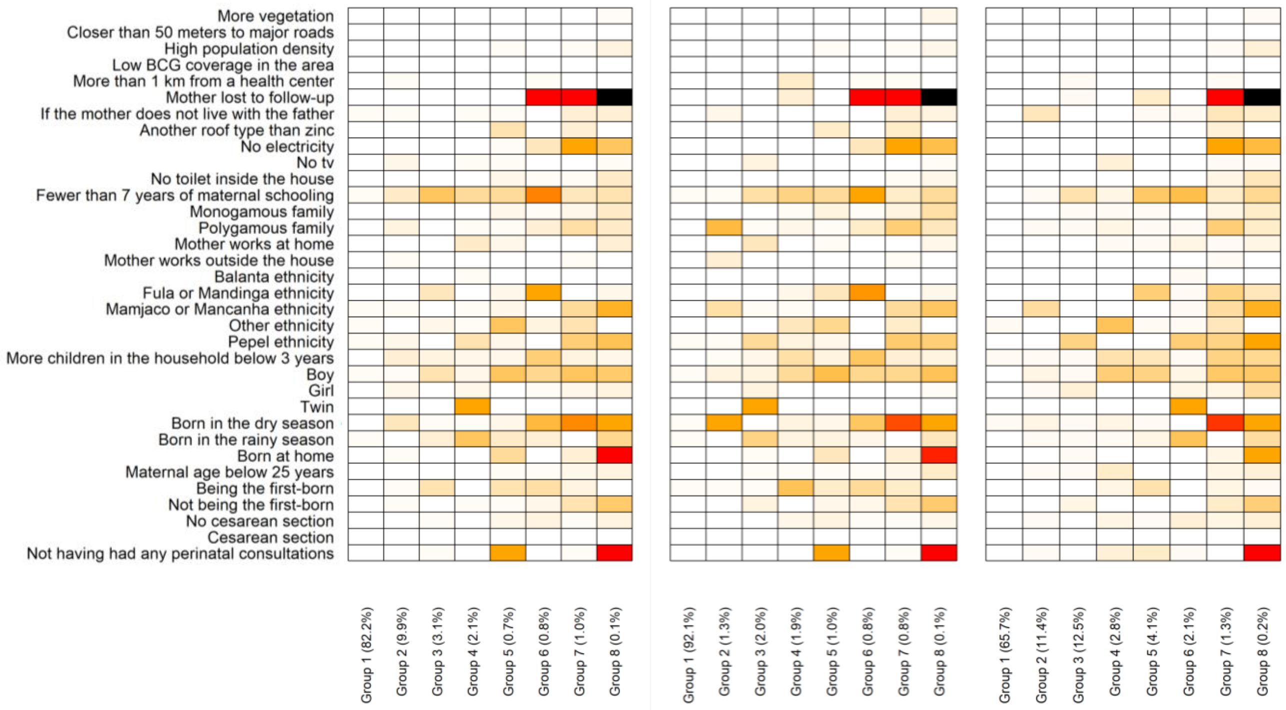

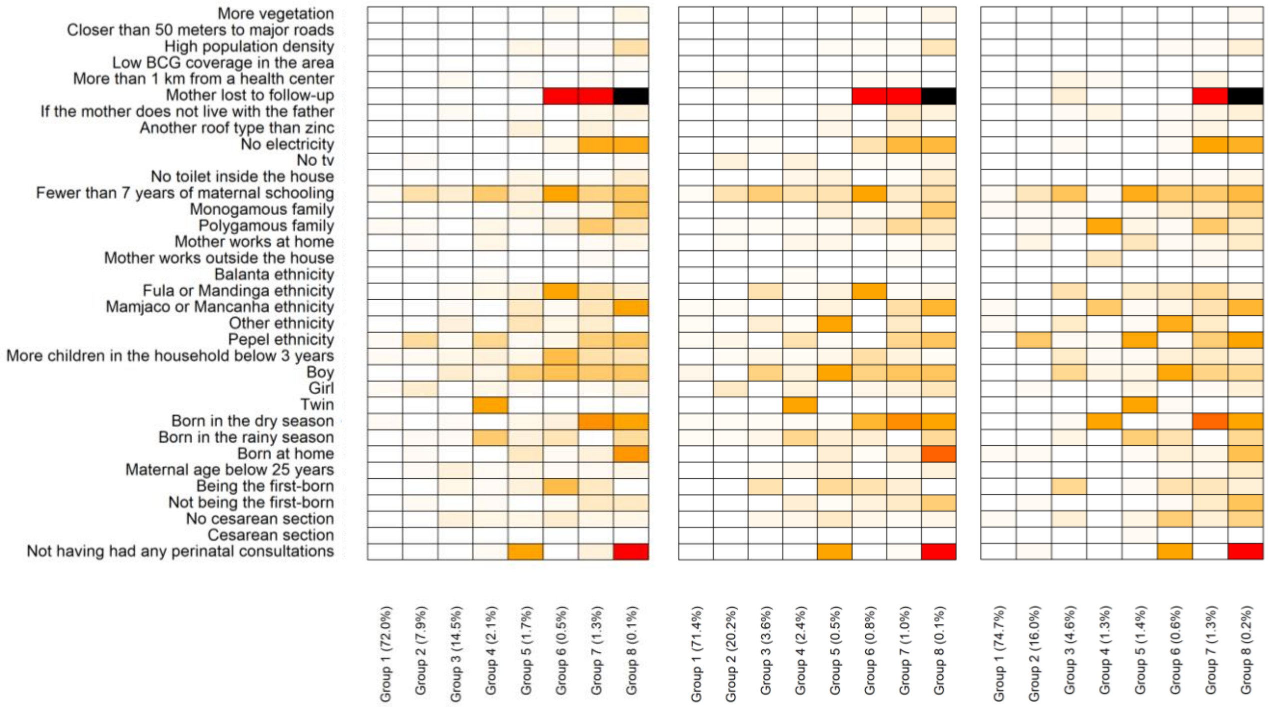

Supplement: Multimedia Appendix 3 [file publichealth_v10i1e48060_app3.pdf]
