# Supplementary material for: Discovering Subgroups of Children With High Mortality in Urban Guinea-Bissau: Exploratory and Validation Cohort Study
Source: JMIR Public Health Surveill. 2024 Apr 9;10:e48060. doi: 10.2196/48060 (PMC11040440; doi:10.2196/48060)
Supplement: Multimedia Appendix 4 [file publichealth_v10i1e48060_app4.pdf]

Multimedia Appendix 4

This multimedia appendix covers the analysis regarding the birth season and polygamous families.

The estimates are robust to the IPCW, as the unweighted estimates were 1.6 (95% CI 0.1-3.7) and 2.9 (95% CI 1.2-4.7) respectively for the hypothesis-generating dataset and temporal validation dataset. The following analyses were conducted on the full data (hypothesis-generating data and temporal validation data) to increase the power for detecting patterns. The rink difference is accelerated from baseline and continues to increase throughout follow-up where the reference decrease after 1 year of age (below figure to the left). The association does not seem to be calendar year dependent and with variation between each birth year (below figure to the right).

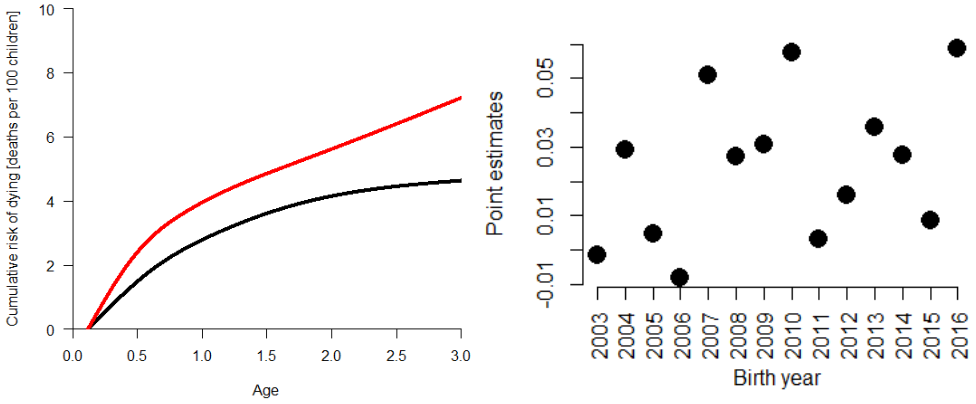

It is especially the ethnical groups Manjaco and Mancanha for which the association is observed (below table).

Calendar-adjusted risk difference		Children born in the dry season and in polygamous families compared with the rest within the ethnical group
Ethnic group	Balanta	1.5 (95% CI -1.7 to 4.8)
	Fula/Mandinga	2.6 (95% CI 1.1-4.2)
	Manjaco/Mancanha	4.7 (95% CI 1.1-7.3)
	Pepel	2.2 (95% CI 0.3-4.1)
	Others	0.8 (95% CI -1.6 to 3.2)

When looking for the geographical distribution in sliding windows of 1000 metres ▪ 1000 metres, the increased risk of being born in the dry season and in polygamous families when assessed only within the sliding window highlights the eastern part of the HDSS area:

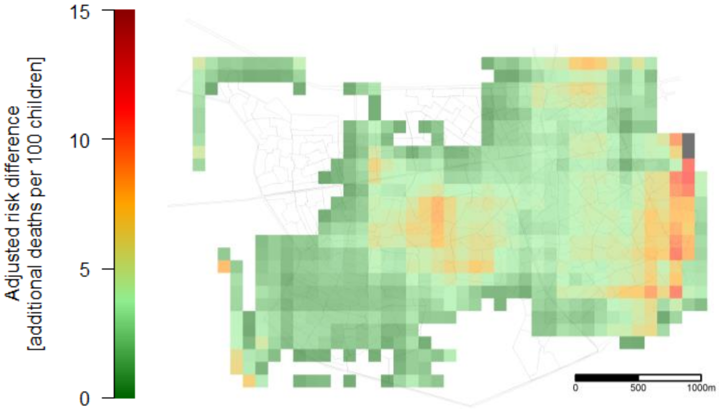

The association was adjusted for crowding (other children below 3 years of age in the household) as it was adjusted for in all TMLE analyses. However, when stratified by crowding across both cohorts, the aMRD was 2.1% (95% CI 0.6%; 3.5%) in the strate of children with other children below 3 years of age in the household, while it was -0.1% (95% CI -2.8%; 2.5%) in the strata without other children below 3 years of age.
